# Evaluation of the Antimicrobial Effectiveness and the Effect of Dosage and Frequency of Sugar-free Chewing Gums on *Streptococcus mutans* Count: An *in vivo* Microbiological Study

**DOI:** 10.5005/jp-journals-10005-1077

**Published:** 2011-04-15

**Authors:** Mohita Marwaha, Manohar Bhat

**Affiliations:** 1 Senior Lecturer, Department of Pedodontics and Preventive Dentistry, SGT Dental College and Research Institute, Gurgaon, Haryana, India; 2 Professor and Head, Department of Pedodontics and Preventive Dentistry, Jaipur Dental College and Hospital, Jaipur, Rajasthan, India

**Keywords:** Sugar-free gums, Chewing gums, *Streptococcus mutans.*

## Abstract

The aim of the present study was to evaluate the antimicrobial efficacy of sugar-free chewing gums and also to assess the effect of dosage and frequency of intake of sugar-free gums on *Streptococcus mutans* count.

**Method :** The sample consisted of 30 subjects, divided into two groups AI and AII. Each group consisted of 15 subjects. Group AI chewed two sugar-free chewing gum, twice daily for 20 minutes (Total four gums daily) and group AII chewed two sugar-free chewing gum, four times daily for 20 minutes (Total eight gums daily) and saliva sample was collected and agar plates were inoculated for *Streptococcus mutans* colony count. The study was carried for a week’s time and saliva samples collected were baseline, day 1 morning and evening, day 4 evening, day 7 morning and evening.

**Results :** After the gum was chewed, it was observed that the colony count started to reduce when compared with baseline in both the groups. The fall in *Streptococcus mutans* count was statistically highly significant with p < 0.001 in both the groups. When comparing between group AI (dosage 4 gums daily) and group AII (dosage 8 gums daily), the fall in *Streptococcus mutans* count for both the groups was not statistically significant with p > 0.05. It was concluded that there was reduction in the level of salivary *Streptococcus mutans,* but was not statistically significant by increasing the dosage and frequency of intake of sugar-free chewing gums.

Therefore, we recommend that dosage of sugar-free chewing gums can be restricted to four gums instead of eight gums per day.

## INTRODUCTION

*Streptococcus mutans* is strongly associated with caries in humans^1^ and its level in the mouth can be a good indicator of caries-risk. However, caries is a multifactorial disease and the presence of high levels of *Streptococcus mutans* at a particular site does not imply that such a site will inevitably develop a lesion. In addition, *Streptococcus mutans* is not found alone in association with caries.

Human oral microorganisms, and specifically *Streptococcus mutans,* do not have enzymes to utilize xylitol as a source of energy for acid production or for synthesis of extracellular polysaccharides.

In 1975, the first xylitol chewing gum was launched almost simultaneously in Finland and United States. The consumption of xylitol, a sugar alcohol of pentitol type, had been found in early 1970s to reduce the incidence of dental caries.

In recent years, the use of chewing gums has increased. Human beings have been chewing gum-like substances since ancient time. The pleasure of chewing is to clean the mouth or freshen the breath. In 1991, Chewing gum was approved as a term for a pharmaceutical dosage form by the commission of European communities.

Chewing gum^2^ is defined as a “solid preparation with a base consisting of gum which should be chewed and not swallowed, providing a slow steady release of medicine contained.”

## BENEFICIAL EFFECTS OF SUGAR-FREE GUM^3^

1. Xylitol and Sorbitol have been used extensively to sweeten chewing gum. Neither sugar substitute is fermented to form acids by oral microorganisms at a rate comparable to that for conventional dietary mono-and disaccharides. Both are ‘caloric sweeteners’ and are capable of being metabolized in the liver, although their uptake from the gut is relatively slow, leading to reduced calorific value and a potential to cause osmotic diarrhea in large doses. In doses expected even in heavy consumers of gum, laxative effects are unlikely.
2. Both sugar substitutes, when added to a chewing gum, elicit a gustatory reflex, which in conjunction with chewing reflexes results in an increase in salivary output in normal individuals. This stimulation peaks in the first few minutes of chewing, and falls to a plateau thereafter due to mainly the loss of taste but also to a softening of the residual gum base. Xylitol has the same sweetness as Sucrose on a weight basis, and in appropriate formulations its negative heat of solution gives rise to a pronounced cooling giving a refreshing effect in the mouth as it dissolves.
3. Sorbitol is not metabolized at all by most oral organisms, it is capable of being fermented by some species, notably those of the mutans group of streptococci.
4. Xylitol does not appear to be metabolized to form acid by oral microorganism, and indeed *in vitro* it is an antimetabolite since on entering cell it is phosphorylated to form an inhibitory compound. Xylitol in chewing gum reduces the proportions of mutans streptococci in plaque or saliva and the amount of plaque present.

Many researches have been done showing the reduction in the salivary levels of *Streptococcus mutans* after the use of chewing gums. Walter J Loesche et al (1984)^4^ concluded that the chewing of xylitol chewing gum for four weeks was associated with 90% reduction in salivary levels of *Streptococcus mutans* where there was little change in salivary *Streptococcus mutans* levels associated with chewing the sorbitol or fructose gums. The chewing of xylitol gums was associated with 60% reduction in the levels of *Streptococcus mutans* in plaque, whereas the chewing of sorbitol or fructose gums had little effect on the levels of *Streptococcus mutans* in plaque.

Therefore, keeping in mind these facts about sugar-free chewing gums, we undertook a study with the following aims and objectives:

1. To evaluate the antimicrobial efficacy of sugar-free chewing gums.
2. To assess the effect of dosage and frequency of intake of sugar-free chewing gums on *Streptococcus mutans* count.

## MATERIALS AND METHODS

Total sample size of 30 subjects was selected between the age group of 6 to 12 years, irrespective of sex and socioeconomic status. The subjects volunteered to participate after verbal and written information. Ethical clearance and informed consent were taken.

### Materials and Equipments

Mouth mirror, explorer, sterile gloves, ultrasonic scaler, sugar-free chewing gums (Orbit), sterile sample collection bottles, ice box filled with ice, transport media, mutans sanguis agar media (HiMEDIA), agar plates, inoculation rod, incubator, etc.

### Subject Selection Criteria

- Subjects between the age of 6 to 12 years
- Systemically healthy subjects
- No history of antibiotic therapy within previous three months
- No fixed or removable orthodontic appliance or removable prosthesis
- No use of any regular or habitual use of sugar-free containing products
- No history of oral prophylaxis done at least three months prior to the study.

After selection, oral prophylaxis of all the subjects was done using ultrasonic scaler. Then the subjects were instructed to abstain from any oral hygiene measures for next 24 hours.

*Day 1 Morning:* Baseline saliva sample was collected by spitting method in sterile sample collecting bottles for all the subjects. Subjects were then divided into two major groups: Group AI and Group AII.

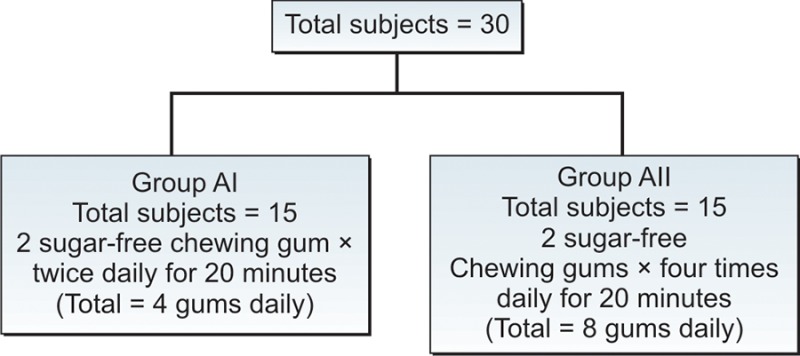



All the chewing gums were given before meals for all the groups. The study was conducted for a week’s time. After collecting baseline samples, the subjects were given the respective chewing gums as per the groups and were asked to chew as instructed under supervision and the saliva sample were again collected after 10 minutes. The subjects were then asked to start maintaining their oral hygiene as regular. The same procedure was repeated on day 1 evening. All the 7 days, the same procedure was followed for all the groups under supervision and the sample collection was taken under aseptic conditions.

### Days of Sample Collection

Day 1: Baseline, morning and evening; day 4: evening; day 7: morning and evening.

The sample was collected in sterile sample bottles and carried in the ice box containing ice (used as transport media) to microbiology laboratory where the culture plates were inoculated for the salivary *Streptococcus mutans* counts.

### Microbiological Procedure and Method of Inoculation

The Mutans-Sanguis agar plates were dried in the incubator for 20 minutes at a temperature of 37°C in an aerobic chamber. Then after drying of the plates, they were labeled and the salivary sample was inoculated on agar plates. The sample was taken in a loop of inoculating rod of diameter 1/1000 CFU/ml and was carried onto the agar plates and strains were made on the plate..

The plates were then kept in incubator for 48 hours at 37°C for the growth of *Streptococcus mutans* colony. After 48 hours, the plates were removed from incubator. *Streptococcus mutans* count and colony count was done manually using magnifying lens. Colonies of *Streptococcus mutans* appeared rough, heaped, irregular resembling frosted glass-white, grey or yellow in color and 0.5 to 2 mm in diameter.

## RESULTS

The data obtained was statistically analyzed by using student’s paired t-test and the following results were obtained.

In Table 1 group AI—mean and standard deviation of salivary *Streptococcus mutans* at baseline was 148.33 ± 66.15 CFU/ml and after chewing the gum on 1st day morning, the count was reduced to 93.67 ± 44.33 CFU/ml and in the evening, the count was observed to be 76.67 ± 37.27 CFU/ml and on 4th day evening, the count was 59.33± 29.37 CFU/ml. On 7th day morning and evening, again decrease in the mutans level was observed, i.e. 52.33 ± 22.65 and 50.67 ± 25.22 CFU/ml respectively.

In group AII—mean and standard deviation of salivary *Streptococcus mutans* at baseline was 156.67 ± 44.22 CFU/ ml and after chewing the gum it was observed to be 120.67 ± 37.10 CFU/ml in the morning and in the evening to 90.00± 29.14 CFU/ml. On 4th day evening, the levels reduced to 70.00 ± 18.80 CFU/ml and on 7th day morning and evening, it was observed to be 67.67 ± 19.82 and 53.33 ± 18.94 CFU/ml respectively.

In Table 2 depicts mean change for salivary *Streptococcus mutans* levels (CFU/ml) for group AI from baseline was 54.67 ± 42.99 CFU/ml on day 1 morning and the mean change on day 1 evening was 71.67 ± 50.20 CFU/ml. The fall in *Streptococcus mutans* count was statistically highly significant p < 0.001. The mean change on day 4 evening 89.00 ± 56.92 CFU/ml, and on day 7 morning and evening, the count was 96.00 ± 61.82 and 97.67 ± 62.27 CFU/ml respectively. The fall in *Streptococcus mutans* count was statistically highly significant with p < 0.001.

In Table 3 depicts mean change for salivary *Streptococcus mutans* levels (CFU/ml) for group AII from baseline was 36.00 ± 19.10 CFU/ml on day 1 morning and the mean change on day 1 evening was 66.00 ± 24.94 CFU/ml . The fall in *Streptococcus mutans* count was statistically highly significant p < 0.001. The mean change on day 4 evening 86.67 ± 38.85 CFU/ml. The mean change on day 7 morning and evening, the count was 89.00 ± 36.11 and 103.33 ± 37.21 CFU/ml respectively. The fall in *Streptococcus mutans* count was statistically highly significant with p < 0.001.

In Table 4 depicts the correlation between the dosage of sugar-free gums with the reduction in the levels of *Streptococcus mutans* count. Although in AII group, the reduction is slightly more as compared to AI group, the difference is not statistically significant with p > 0.05.

**Table 1:** **Table 1:** Mean and standard deviation values of salivary *Streptococcus mutans* (in CFU/ml), at various levels of group AI, AII

*Group*		*Level*											
		*Basal value*		*1st day morning*		*1st day evening*		*4th day evening*		*7th day morning*		*7th day evening*	
Group AI		148.33 ± 66.15		93.67 ± 44.33		76.67 ± 37.27		59.33 ± 29.37		52.33 ± 22.65		50.67 ± 25.22	
Group AII		156.67 ± 44.22		120.67 ± 37.10		90.00 ± 29.14		70.00 ± 18.80		67.67 ± 19.82		53.33 ± 18.94	

**Table 2:** **Table 2:** Statistical comparison of mean change and standard deviation (by Student’s t-test, paired) of salivary *Streptococcus mutans* (CFU/ml) observed from baseline value to various levels of group AI

*Group AI*		*Level*											
		*1st day morning*		*1st day evening*		*4th day evening*		*7th day morning*		*7th day evening*	
Mean change + SD		54.67 ± 42.99		71.67 ± 50.20		89.00 ± 56.92		96.00 ± 61.82		97.67 ± 62.27	
p-value		< 0.001		< 0.001		< 0.001		< 0.001		< 0.001	
Significance		HS		HS		HS		HS		HS	

HS: High significance

## DISCUSSION

The surface of the oral cavity is constantly colonized by microorganisms. One milliliter of whole saliva may contain more than 200 million organisms representing more than 250 different species.

Streptococcus constitutes an essential part of the microflora, which constantly colonize the mucous membrane and the teeth. The microorganisms are regularly digested when saliva is swallowed and there is a fluctuating amount within the oral cavity because the microbial deposits building upon mucous membrane and, in particular, on tooth surface grow and multiply, and thus provide a reservoir for oral environment.

*Streptococcus mutans* is a gram positive, facultative anaerobic bacteria commonly found in the human oral cavity and is a significant contributor to tooth decay. The microb was first described by Clarke in 1924.

Along with *Streptococcus sobrinus, Streptococcus mutans* play a major role in tooth decay, metabolizing sucrose to lactic acid. The environment created in the mouth by this process is what cause the highly mineralized tooth enamel to be vulnerable to decay. Sucrose is utilized by *Streptococcus mutans* to produce a sticky, extracellular dextran based polysaccharide that allows them to coadhere to each other forming plaque.

*Streptococcus mutans* produces dextran via the enzyme dextran-sucrose using sucrose as a substrate. Sucrose is only sugar that *Streptococcus mutans* can use to form this sticky polysaccharide. It is combination of plaque and acid that leads to dental decay.

Human oral microorganisms, and specifically *Streptocoocus mutans,* do not have enzymes to utilize xylitol as a source of energy for acid production or for synthesis of extracellular polysaccharides.

Sweetness of xylitol is similar to that of sucrose, the sweet taste appears and disappears a little faster and rapid dissolution of xylitol in water results in a cool feeling in the mouth.^5^

**Fig. 1:**
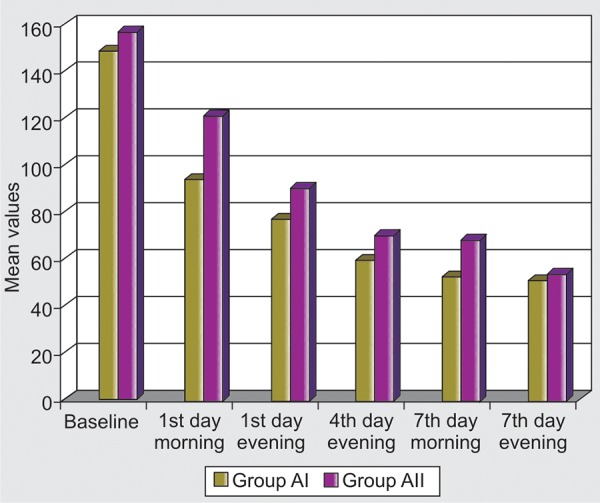
Mean values of salivary *Streptococcus mutans* (CFU/ ml), at various intervals of two sugar-free chewing gum groups (group AI, group AII)

**Table 3:** **Table 3:** Statistical comparison of mean change and standard deviation (by student’s t-test, paired) of salivary *Streptococcus mutans* (CFU/ml) observed from baseline value to various levels of group AII

*Group All*		*Level*											
		*1st day morning*		*1st day evening*		*4th day evening*		*7th day morning*		*7th day evening*	
Mean change ± SD		36.00 ± 19.10		66.00 ± 24.94		86.67 ± 38.85		89.00 ± 36.11		103.33 ± 37.21	
p-value		< 0.001		< 0.001		< 0.001		< 0.001		< 0.001	
Significance		HS		HS		HS		HS		HS	

HS: High significance

**Table 4:** **Table 4:** Statistical comparison (by unpaired t-test) of salivary *Streptococcus mutans* (CFU/ml) of mean change at various levels between group AI and group AII

*Group*		*Level*											
		*1st day morning*		*1st day evening*		*4th day evening*		*7th day morning*		*7th day evening*	
Group AI		54.67 ± 42.99		71.67 ± 50.20		89.00 ± 56.92		96.00 ± 61.82		97.67 ± 62.27	
Group AII		36.00 ± 19.10		66.00 ± 24.94		86.67 ± 38.85		89.00 ± 36.11		103.33 ± 37.21	
p-value		> 0.05		> 0.05		> 0.05		> 0.05		> 0.05	
Significance		NS		NS		NS		NS		NS	

NS: Not significance

A large portion of ingested xylitol is directly absorbed by small intestine and subsequently metabolized. The remainder reaches the large intestine where it is fermented by enterobacteria. The available energy value of xylitol as a nutrition indicator is 3 kcal/gm. Metabolism of xylitol is insulin independent and it produces less of a laxative effect than either sorbitol or mannitol, probably due to its high rate of absorption (72 to 95%). Most people can tolerate 300 gm of xylitol in a single dose and daily doses as high as 200 gm causes laxative symptoms only rarely.

Albert Bar (1988)^6^ suggested mechanisms for caries inhibitory effect of xylitol:

- Xylitol due to its organoleptic properties, stimulates salivation, thereby increases plaque pH, and thus promotes remineralization.
- Other specific effects of xylitol on the composition of saliva have been proposed, such as increase in the concentration of basic amino acids and glycine.
- Increase in total salivary protein in parotid and palatine gland secretions.
- Higher activities of amylase.
- Higher activities of carbonic anhydrase, lactoperoxidase and invertase like enzymes as well as elevated levels of thiocyanate ions.

All these changes indicate a xylitol mediated enhancement of natural defense mechanisms against caries in terms of a better buffering capacity and a higher bacteriostatic activity of saliva.

Xylitol directly inhibits demineralization of enamel, probably by interfering with the transport of dissolved hydroxyapatite from the lesion to the bulk solution by lowering the diffusion coefficients of calcium and phosphate ions.

Debra Simons (1996)^7^ suggested that the effects of xylitol in chewing gum can be explained by various phenomena:

1. The reduction in sucrose intake produced by dietary substitution with a nonfermentable carbohydrate.
2. The sweet taste of xylitol, stimulates the salivary flow with an attendant increase in buffer capacity and defense factors.
3. Xylitol is associated with increased soluble calcium in plaque, which may assist in remineralization of enamel.
4. Xylitol also possesses an antibacterial property reportedly due to its entering the bacterial cell wall via the fructose phosphotransferase system. The xylitol 5-phosphate thus formed, inhibits bacterial growth partly by establishing an energy-consuming futile cycle and partly by inhibiting glucose uptake and metabolism.

In our study: In Group AI—the mean and standard deviation of salivary *Streptococcus mutans* at baseline was 148.33 ± 66.15 CFU/ml and after chewing the gum on 1st day morning, the count was reduced to 93.67 ± 44.33 CFU/ml and in the evening, the count was observed to be 76.67 ± 37.27 CFU/ml and on 4th day evening, the count was 59.33 ± 29.37 CFU/ml. On 7th day morning and evening, again decrease in the mutans level was observed, i.e. 52.33 ± 22.65 and 50.67 ± 25.22 CFU/ml respectively.

In the present study, we observed that there is a reduction in salivary *Streptococcus mutans* count after chewing of sugar-free containing gums, which goes in accordance with Pernilla Lif Holgerson, Inger Sjostrom, Christina Stecksen-Blicks, Svante Twetman (2007)^8^ who concluded the same, and the possible reason attributed for the reduction in *Streptococcus mutans* count could be the xylitol in chewing gum as it possesses an antibacterial property due to its entrance into the bacterial cell wall via the fructose phosphotransferase system. The xylitol 5- phosphate thus formed, inhibits bacterial growth partly by establishing an energy-consuming futile cycle and partly by inhibiting glucose uptake and metabolism (Debra Simons 1996)^7^.

Group AII—mean and standard deviation of salivary *Streptococcus mutans* at baseline was 156.67 ± 44.22 CFU/ ml and after chewing the gum it was observed to be 120.67 ± 37.10 CFU/ml in the morning and in the evening to 90.00± 29.14 CFU/ml. On 4th day evening, the levels reduced to 70.00 ± 18.80 CFU/ml and on 7th day morning and evening, it was observed to be 67.67 ± 19.82 and 53.33 ± 18.94 CFU/ml respectively.

The result of the our investigation suggest that there is a reduction in salivary *Streptococcus mutans* count which goes in accordance with Wennerholm K, CG Emilson (1989)^9^, Pernilla Lif Holgerson, Inger Sjostrom, Christina Stecksen-Blicks, Svante Twetman (2007)^8^ who evaluated that chewing of xylitol containing gums caused a significant reduction in salivary *Streptococcus mutans* count and amount of dental plaque formation.

In group AI from baseline was 54.67 ± 42.99 CFU/ml on day 1 morning and the mean change on day 1 evening was 71.67 ± 50.20 CFU/ml . The fall in *Streptococcus mutans* count was statistically highly significant p < 0.001.

The result of our investigation suggest that there is a reduction in *Streptococcus mutans* count in saliva, which goes in accordance with the study conducted by Hildebrandt and Sparks (2000),^10^ KK Makinen, Kauko P Isotupa, Pirkko-Liisa Makinen, E Soderling (2005),^11^ and S Harosaku et al (2007)^12^ who concluded the same.

In Group AII from baseline was 36.00 ± 19.10 CFU/ml on day 1 morning and the mean change on day 1 evening was 66.00 ± 24.94 CFU/ml. The fall in *Streptococcus mutans* count was statistically highly significant p < 0.001. The mean change on day 4 evening 86.67 ± 38.85 CFU/ml. The mean change on day 7 morning and evening, and the count was 89.00 ± 36.11 and 103.33 ± 37.21 CFU/ml respectively. The fall in *Streptococcus mutans* count was statistically highly significant with p < 0.001.

The result of our investigation suggests that the saliva levels of *Streptococcus mutans* are decreased in subjects with sugar-free chewing gum intake.

P Milgrom et al (2006)^13^ have also reported reduction in *Streptococcus mutans* in both saliva and plaque when a dosage of 12 pellets gums/day was given.

In our study when correlating between the dosage of sugar-free gums and the level of reduction of *Streptococcus mutans* count, although in AII group, the reduction is slightly more as compared to AI group, the difference is not statistically significant with p > 0.05.

As we were not aware of the exact concentration of xylitol and other artificial sweeteners present per gum, therefore, we have calculated the dosage based on the number of gums consumed per day. Hence, in our study no direct relationship was observed between dosage and reduction of *Streptococcus mutans* counts. Therefore, we recommend that dosage of sugar-free gums can be restricted to four chewing gums per day instead of eight.

## CONCLUSION

1. In antibacterial efficacy, sugar-free chewing gums have shown reduction in salivary *Streptococcus mutans* count, and therefore it can be used as an alternative means.
2. When comparing between intake of sugar-free chewing gums twice a day and four times a day, equal reduction in *Streptococcus mutans* count was observed.
3. Finally, based on our study, sugar-free chewing gums has shown significant reduction in salivary *Streptococcus mutans* count, and therefore we recommend that two sugar-free chewing gums twice daily (total dosage— 4 gums per day) is sufficient enough to reduce the colonization of *Streptococcus mutans* in oral cavity.

Further studies can be conducted with larger sample size and also to see its effect on any other microorganisms directly or indirectly associated with dental diseases.
